# Structure of the HCMV UL16-MICB Complex Elucidates Select Binding of a Viral Immunoevasin to Diverse NKG2D Ligands

**DOI:** 10.1371/journal.ppat.1000723

**Published:** 2010-01-15

**Authors:** Steffen Müller, Georg Zocher, Alexander Steinle, Thilo Stehle

**Affiliations:** 1 Interfaculty Institute for Biochemistry, University of Tuebingen, Tuebingen, Germany; 2 Department of Immunology, Interfaculty Institute for Cell Biology, University of Tuebingen, Tuebingen, Germany; 3 Department of Pediatrics, Vanderbilt University School of Medicine, Nashville, Tennessee, United States of America; Harvard Medical School, United States of America

## Abstract

The activating immunoreceptor NKG2D promotes elimination of infected or malignant cells by cytotoxic lymphocytes through engagement of stress-induced MHC class I-related ligands. The human cytomegalovirus (HCMV)-encoded immunoevasin UL16 subverts NKG2D-mediated immune responses by retaining a select group of diverse NKG2D ligands inside the cell. We report here the crystal structure of UL16 in complex with the NKG2D ligand MICB at 1.8 Å resolution, revealing the molecular basis for the promiscuous, but highly selective, binding of UL16 to unrelated NKG2D ligands. The immunoglobulin-like UL16 protein utilizes a three-stranded β-sheet to engage the α-helical surface of the MHC class I-like MICB platform domain. Intriguingly, residues at the center of this β-sheet mimic a central binding motif employed by the structurally unrelated C-type lectin-like NKG2D to facilitate engagement of diverse NKG2D ligands. Using surface plasmon resonance, we find that UL16 binds MICB, ULBP1, and ULBP2 with similar affinities that lie in the nanomolar range (12–66 nM). The ability of UL16 to bind its ligands depends critically on the presence of a glutamine (MICB) or closely related glutamate (ULBP1 and ULBP2) at position 169. An arginine residue at this position however, as found for example in MICA or ULBP3, would cause steric clashes with UL16 residues. The inability of UL16 to bind MICA and ULBP3 can therefore be attributed to single substitutions at key NKG2D ligand locations. This indicates that selective pressure exerted by viral immunoevasins such as UL16 contributed to the diversification of NKG2D ligands.

## Author Summary

Cytotoxic lymphocytes such as natural killer (NK) cells or CD8 T cells have the ability to detect and destroy cells infected by viruses. They therefore are tools on which the human immune system critically depends in order to control viral infections. To avoid discovery by cytotoxic lymphocytes and to allow for longtime persistence in the human host, the human cytomegalovirus (HCMV) has developed a multitude of immune evasive strategies that are mediated by so-called immunoevasins. We present here a structure-function analysis of one of the best-known HCMV immunevasins, UL16, and its interaction with a cellular ligand for NK cells, MICB. The normal function of MICB is to activate NK cells by engaging the most well-known NK receptor, NKG2D. Our results provide molecular evidence for the strategy used by UL16 to disable NK cell activation. In a rare example of structural mimicry that has likely arisen through convergent evolution, UL16 mimics a central binding motif of the structurally unrelated NKG2D protein. This allows UL16 to engage and disable several diverse NKG2D ligands, while others have apparently evolved to escape recognition by UL16 through alteration of key residues at strategic locations.

## Introduction

Human cytomegalovirus (HCMV) is a β-herpesvirus that causes lifelong asymptomatic infections in healthy individuals but endangers the lives of immunocompromised individuals and very young children [1]. Cytotoxic lymphocytes such as CD8 T cells and natural killer (NK) cells are essential for the control of HCMV infection [1]–[4]. HCMV possesses a broad arsenal of immune evasive strategies that counteract cellular immunosurveillance and ensure long-term persistence in infected human hosts [2], [5]–[8]. One such strategy is the degradation of MHC class I molecules in order to subvert presentation of HCMV-derived peptide antigens to CD8 αβ T cells [2],[6],[8]. However, in line with the ‘missing-self-hypothesis’, impaired MHC class I expression results in a decreased engagement of MHC class I-specific inhibitory NK cell surface receptors and thus may facilitate NK cell-mediated lysis of the infected cells [9]. NK cell activity, however, is not solely controlled by receptors that inhibit NK cell activation, but rather is determined by the integration of signals from both inhibitory and activating NK cell receptors [10]–[12]. A potent activating receptor that mediates NK surveillance of stressed cells such as infected or malignant cells (‘induced-self’ or ‘stressed-self’ recognition) is NKG2D (natural-killer group 2, member D) [11],[13],[14]. NKG2D is a C-type lectin-like homodimer expressed on NK cells and cytotoxic T cells [15]. In humans, NKG2D transmits activating (NK cells) or co-stimulatory (CD8 αβ T cells and γδ T cells) signals via the associated DAP10 adaptor [16] and is triggered through engagement of cell stress-inducible MHC class I-related ligands belonging to the diverse MIC (MHC class I chain related molecule) and ULBP (UL16 binding protein) families. Two MIC (MICA and MICB) and six ULBP proteins (ULBP1-6) are currently known [2], [13], [14], [17]–[19]. The ULBP proteins are also sometimes referred to as ‘retinoic acid early transcript’ proteins (RAET; ULBP1/RAET1I, ULBP2/RAET1H, ULBP3/RAET1N, ULBP4/RAET1E, ULBP5/RAET1G and ULBP6/RAET1L). To thwart an antiviral NKG2D-mediated immune response, HCMV counteracts virally induced cellular expression of NKG2D ligands by means of several immunoevasins [2],[5],[8]. HCMV-encoded glycoproteins UL16 and UL142 selectively prevent the surface expression of MICB, ULBP1 and ULBP2 (UL16) and MICA (UL142), respectively, through intracellular retention [2], [20]–[23]. The significance of evasion from NKG2D-mediated immunosurveillance is further highlighted by the recent discovery that the HCMV gene UL112 is transcribed into a microRNA (miRNA) which specifically suppresses translation of MICB mRNA [24]. Although all NKG2D ligands share a MHC class I-like α1α2-platform domain [25]–[28] that binds NKG2D, UL16 does not bind to MICA, ULBP3, ULBP4 or ULBP5 [18], [29]–[31]. This selectivity is surprising since MICA and MICB are highly homologous in sequence (83% identical residues in the α1α2 region) but much more distantly related to the ULBP molecules (that share 21–29% identical residues in the α1α2 region with the MICs and 38–59% amino acid sequence identity among each other), which were originally discovered in a screen for UL16-binding proteins [18],[32].

In order to elucidate the structural basis for the ability of UL16 to engage highly diverse NKG2D ligands and to compare this promiscuous binding mode to that of NKG2D, we determined the structure of the UL16 ectodomain in complex with the α1α2-platform domain of MICB (MICBpf) at 1.8 Å resolution (Table 1). We also expected structural insights into the selective UL16 binding to MICB (but not MICA) and to some ULBP family members as selective pressure exerted by viral immunoevasins such as UL16 may have contributed to the diversification of NKG2D ligands [2],[17],[23]. We find that UL16, which possesses no structural homology to NKG2D, nevertheless employs a NKG2D-like binding mode to interact with MICB. Our results also offer structural explanations for the selective UL16 binding to some NKG2D ligands, and illustrate how the immunological arms race between a persistent pathogen and the human immune system may have driven the evolution of proteins of both, virus and host.

**Table 1:** Data collection and refinement statistics (Molecular Replacement).

	Native
**Data collection**	
Space group	P2_1_2_1_2_1_
No. of complexes in asymmetric unit	2
Unit cell dimensions	
*a*, *b*, *c* (Å)	58.1, 104.2, 146.8
Resolution (Å)	50-1.8
*R*_merge_	7.2 (49.4)a)
*I*/σ*I*	16.9 (3.4)
Completeness (%)	98.7 (97.5)
Redundancy	8.9 (6.9)
Wilson Factor (Å^2^)	24.1
**Refinement**	
Resolution (Å)	50-1.8
No. reflections	
Measured	733339
Unique	82272
R_work_	17.7
R_free_ (test set of 5%)	21.3
No. of non-H atoms	5980
Protein	5084
Carbohydrates	196
PEG8000	49
Acetate	12
Water	639
Average isotropic B factor (Å^2^)	
Protein main chain	28.0
Protein side chain	34.1
Carbohydrates (NAG)	41.5
PEG8000	55.6
Acetate	65.0
Water	39.1
R.m.s deviations	
Bond lengths (Å)	0.006
Bond angles (°)	1.057
Ramachandran regionsb)	
most favorable (%)	97.4
Allowed	2.6
Outliers	0

a) Highest resolution shell is shown in parenthesis. A single crystal was used to assemble the data set.

b) Determined with Coot [49] version 0.5.

## Results

### Structure of UL16

**Figure 1:**
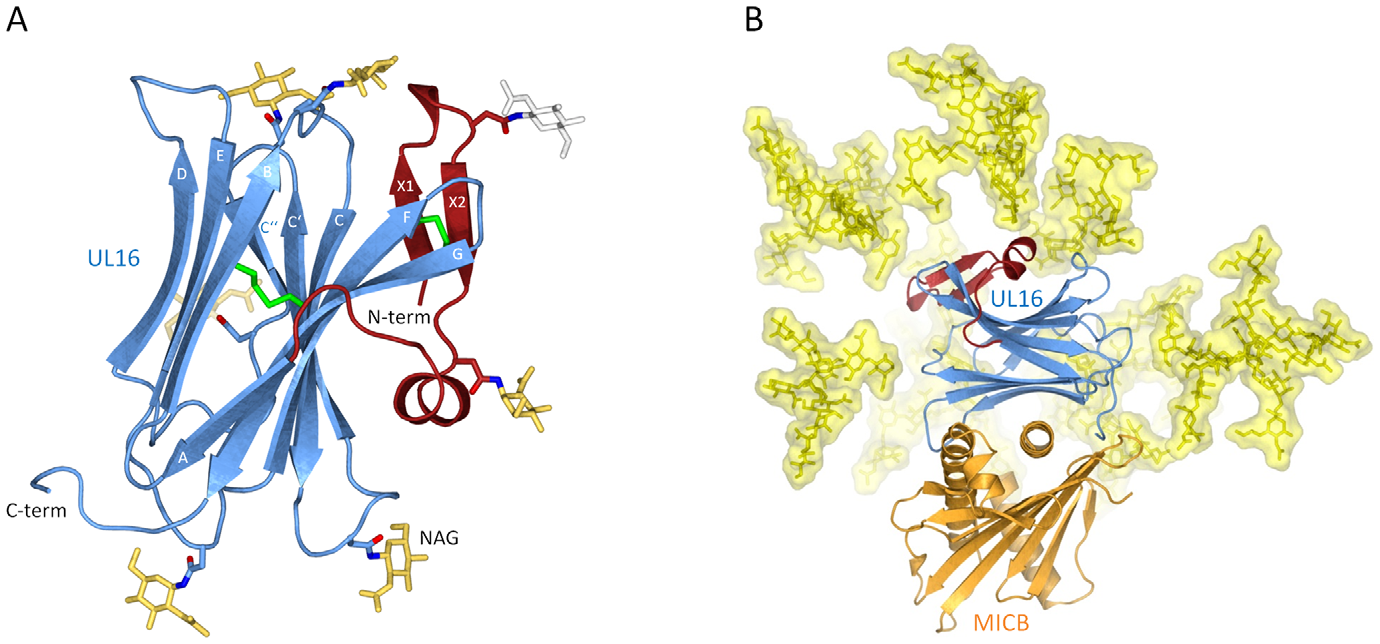
Structure of UL16 in complex with MICBpf. (A), Ribbon drawing of the structure of UL16. The portion of UL16 belonging to the V-type immunoglobin [35] superfamily fold is colored blue, and the N-terminal “plug” is colored red. Glycosylated asparagines (nitrogen atoms dark blue, oxygen atoms red) and attached N-acetylglucosamine residues (yellow) are shown as ball-and-stick models. The grey N-acetylglucosamine residue attached to Asn35 has high temperature factors and was therefore not included in the refinement. Disulfide bonds are shown in green. (B) Structure of the UL16-MICBpf complex. UL16 is colored as in (A), MICBpf is shown in orange. In order to visualize the native glycosylation of UL16, modeled glycans are shown in yellow as ball-and-stick models with a semitransparent surface. See *Materials and Methods* for details.

UL16 is a heavily glycosylated 50 kDa type I transmembrane glycoprotein whose structure could not be predicted from its primary sequence [33]. In order to obtain soluble and homogeneously glycosylated protein for our structural and surface plasmon resonance (SPR) studies, we expressed the UL16 ectodomain in Chinese hamster ovary (CHO) Lec 3.2.8.1 cells [34]. UL16 was co-crystallized with MICBpf refolded from *E. coli* inclusion bodies (see *Materials and Methods*). The UL16 ectodomain folds into a modified version of the immunoglobulin (Ig)-like domain (Figure 1A). The presence of nine β-strands, arranged in two antiparallel β-sheets (formed by β-strands A, G, F, C, C′, C″ and β-strands D, E, B, respectively) and a central disulfide bond linking β-strands B and F clearly classifies it as a variable (V-type) Ig-like domain [35]–[37]. In contrast to classical V-type Ig domains, however, UL16 also has an additional N-terminal “plug” (amino acids 27–50), formed by a two-stranded antiparallel β-sheet (β-strands X1 and X2) and a short 3_10_-helix (Figure 1A). The plug covers the concave side of the AGFCC′C″ β-sheet and is covalently linked to the Ig-like core with a disulfide bond between β-strands X2 and F. The UL16-MICBpf complex was partially deglycosylated prior to crystallization, leaving only single N-acetylglucosamine (NAG) molecules attached to glycosylation sites. While there is no evidence for O-linked glycosylation, our electron density maps provide clear evidence for the presence of NAGs at seven out of eight putative N-glycosylation sites (asparagines 35, 41, 68, 84, 95, 101 and 132). Modeling experiments show that native glycosylation would effectively shield much of the UL16 surface from solvent (Figure 1B). In particular, the outward-facing AGFCC′C″ β-sheet and the N-terminal plug are expected to be mostly covered with glycans in the fully glycosylated protein. By contrast, the solvent-exposed face of the DEB β-sheet is devoid of glycans and available for interactions with other proteins.

### Structure of MICB

**Figure 2:**
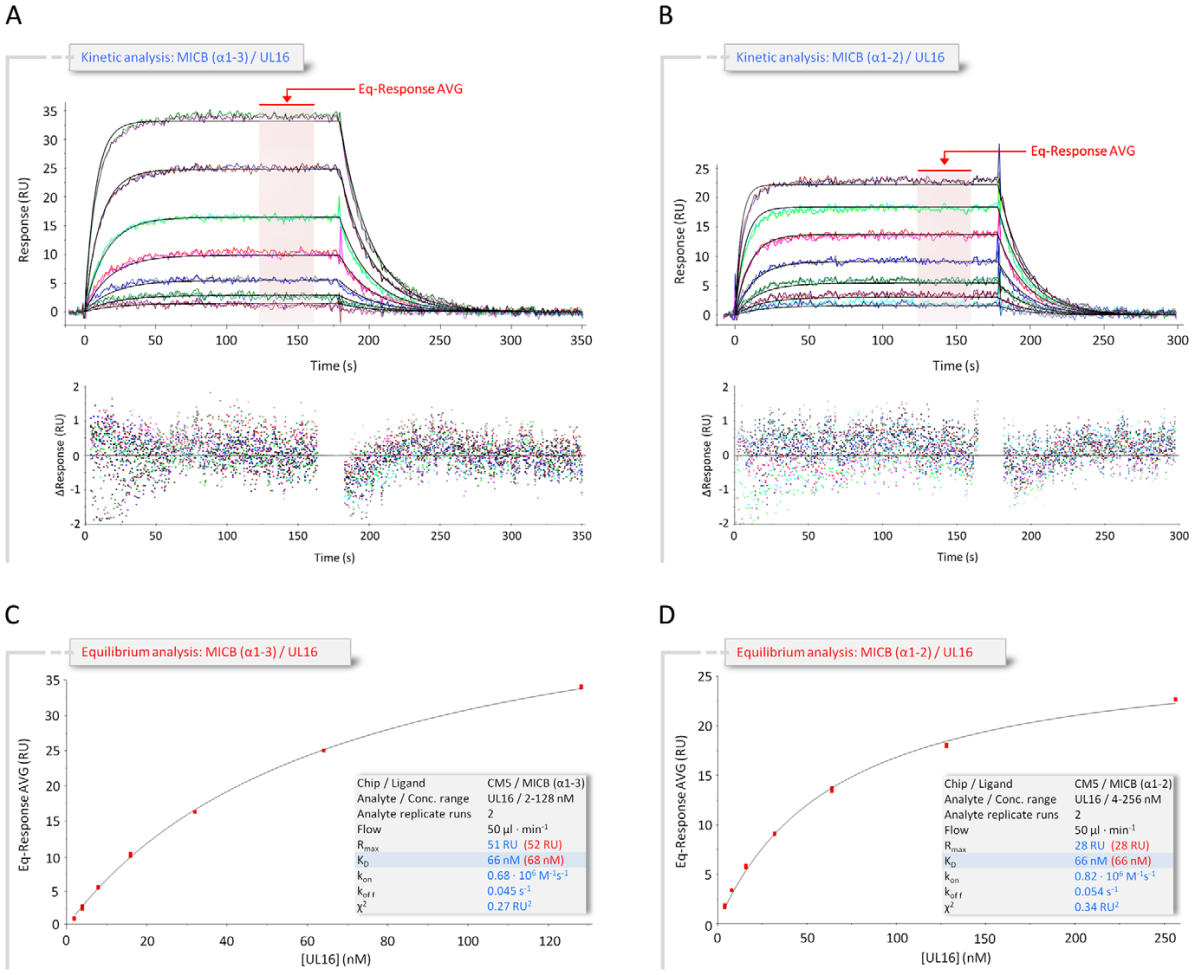
Kinetic and equilibrium SPR analyses of UL16 interactions with MICB. (A,B) Kinetic analyses of UL16 binding to covalently immobilized MICB proteins comprising domains α1 and α2 only (MICBpf) (A), and domains α1, α2 and α3 (B). Each individual analyte concentration was injected twice and data are representative of at least two separate experiments with similar results. Double-referenced sensorgrams (shown in color) are overlaid with fits of a “1∶1 binding with mass transfer” model (black lines). Corresponding residual plots below the sensorgrams show the kinetic-fit range and absolute deviation (Δ) of data points from curve fit values. The red arrow and the red highlighted area of the sensorgram series indicate data used to determine averaged (AVG) equilibrium (Eq) response values (Eq-Response AVG) for equilibrium analysis. (C,D) Equilibrium analysis of UL16 binding to MICBpf (C) and MICB domains α1, α2 and α3 (D). Averaged equilibrium response values (red squares) are plotted against injected UL16 concentrations and fitted to a “1∶1 Langmuir isotherm” model (black line). The shaded boxes contain additional information about setup details (black font) and measured parameters from kinetic (blue font) and equilibrium analysis (red font).

**Figure 3:**
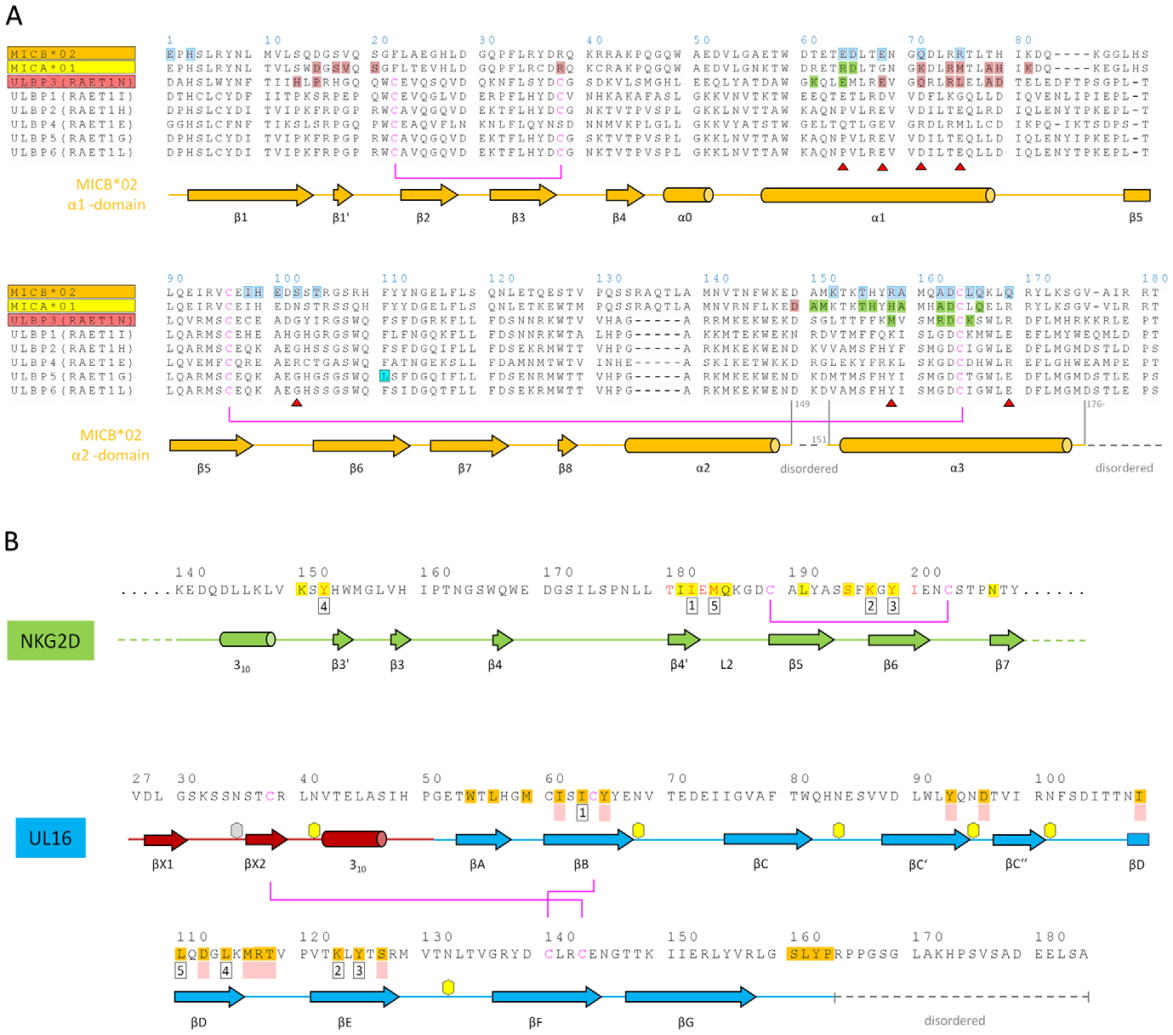
Amino acid sequences of NKG2D ligands, NKG2D, and UL16. (A) Sequence alignment of NKG2D ligands. Sequences of the α1α2-platform domains of NKG2D ligands MICA*01, MICB*02, ULBP1, ULBP2, ULBP3, ULBP4, ULBP5 and ULBP6 are included in the alignment. The alternative RAET nomenclature of ULBP proteins is indicated. Secondary structure elements as observed in the structure of MICBpf in complex with UL16 were assigned by DSSP [62] and are represented with cylinders (helices) and arrows (β-strands) below the alignment. Helices are named as described in [28]. Residues shaded in blue contact UL16 in the UL16-MICBpf complex. Residues shaded in salmon contact the salmon-colored NKG2D monomer (Figures 5A, B) in the MICA-NKG2D and ULBP3-NKG2D complex structures [26],[28]. Residues shaded in green contact the green NKG2D monomer (Figures 5A, B) in the MICA-NKG2D and ULBP3-NKG2D complex structures. Residues marked with a red triangle indicate substitutions between MICA and MICB in regions that contact UL16 in the MICBpf-UL16 complex. The ULBP5 residue boxed in cyan was recently shown to be the major determinant for diminished binding to NKG2D and UL16 [31]. Disulfide bridges and corresponding cysteines are represented with magenta lines. Gaps are indicated by (−). (B) Structural mimicry of UL16. Shown are relevant portions of the sequences of the green human NKG2D monomer [26],[28] (Figures 5A, B) and UL16. Secondary structure elements as observed in the structure of MICBpf in complex with UL16 and MICA in complex with NKG2D [26], respectively, were assigned and represented as described in panel A. The five residues marked with numbered black boxes below the sequence define the central binding motif that engages MICBpf or, in the case of NKG2D, MICA [26], in a similar manner (Figure 5C). Residues with the same number superimpose in space, although they are located in different regions in the protein sequences. Residues shaded in yellow and orange form contacts with MICA in the case of NKG2D [26] and with MICBpf in the case of UL16, respectively. NKG2D residues in red contact ULBP3 in the ULBP3-NKG2D complex [28]. Residues that augment the central binding motif, performing similar functions in the UL16-MICBpf and NKG2D-MICA complexes are marked with filled light red boxes below the sequence. An example is shown in Figure 5C. Disulfide bridges are represented with magenta lines. Hexagons mark the seven UL16 asparagine residues linked with glycans as observed in the UL16-MICBpf complex.

The extracellular region of MICB consists of two structural domains, the α1α2-platform domain (MICBpf) and the C-type Ig-like α3-domain [25]. The α3-domain is present only in the MIC family members of NKG2D ligands, but not among members of the ULBP family [17], [25]–[28]. Our SPR measurements (Figure 2 and Table S1) yielded almost identical dissociation constants (K_D_) for the complexes formed by UL16 with MICBpf (K_D_ = 66 nM) or the complete MICB ectodomain (K_D_ = 67 nM), respectively. Together with a previous report [29], this demonstrates that the α3-domain does not contribute to UL16 binding. Based on these results, only MICBpf was expressed and used for co-crystallization with UL16. As previously reported for the unliganded MICB [25], MICBpf folds into a structure that closely resembles MHC class I molecules, with two long parallel α-helices, contributed by domains α1 and α2, arranged above an eight-stranded antiparallel β-sheet (Figure 1B; for nomenclature of domains and secondary structure elements see Figure 3A). Comparison of MICBpf with the structure of the unliganded MICB ectodomain [25] shows that the platform domain remains essentially unchanged upon engagement of UL16 (root-mean-square deviation of 1.4 Å for 172 common Cα atoms). Although minor differences are seen within three surface-exposed loops and a short N-terminal helix (α0), the residues in these regions have elevated temperature factors and do not contact UL16.

### The UL16-MICB interface

**Figure 4:**
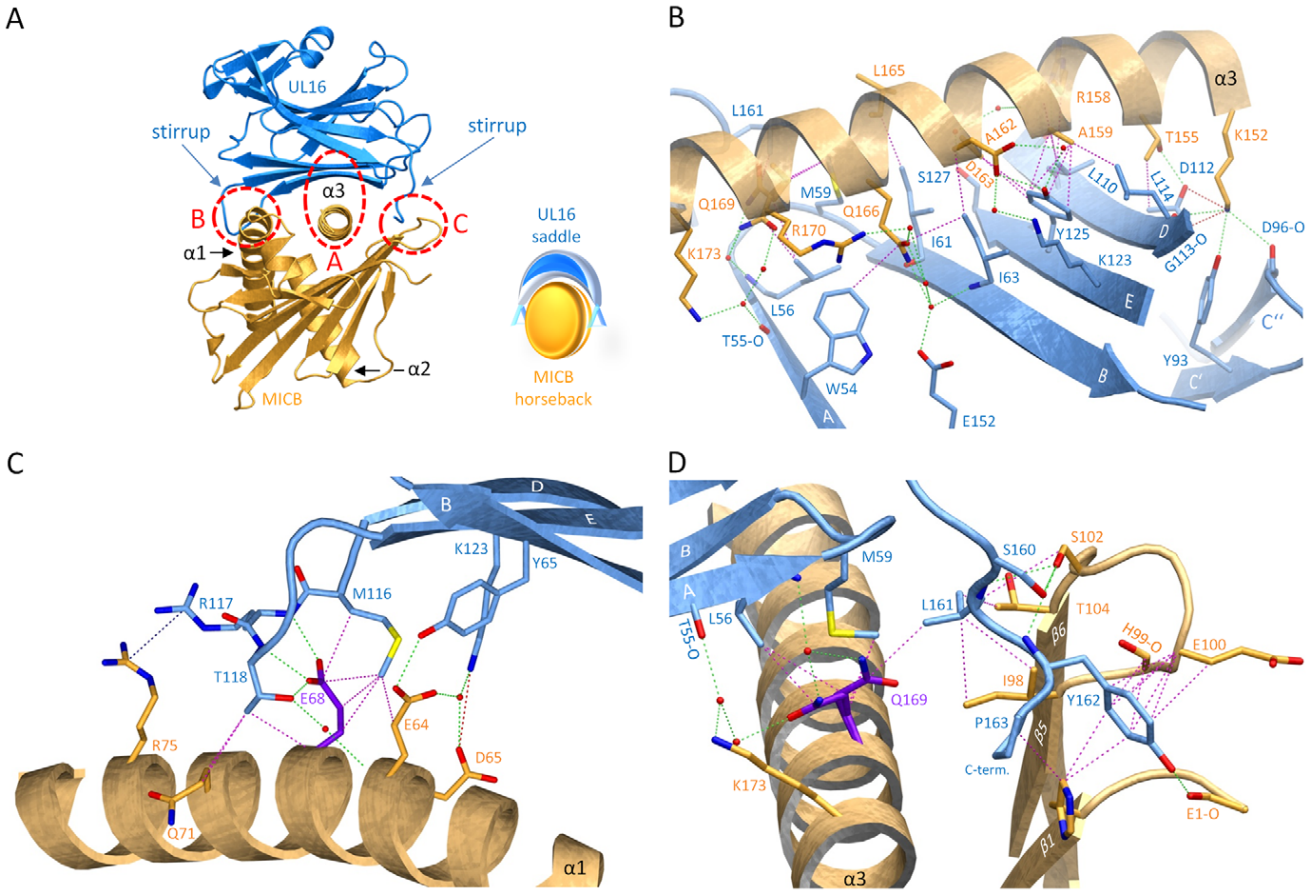
Interaction between UL16 and MICBpf. (A), Ribbon tracing of the complex using the color code from Figure 1. Also shown in the lower right corner of panel A is a schematic representation of the “saddle on horseback” arrangement between UL16 and MICB. (B–D), The three major contact regions A, B and C of the complex. Nitrogen, oxygen and sulfur atoms are colored blue, red, and yellow, respectively. Hydrogen bonds and salt bridges are represented with dashed green and red lines, respectively, and hydrophobic contacts (distance<4.0 Å) are shown as dashed magenta lines. The dashed blue line indicates π-π interactions of two arginine guanidinium groups. Water molecules are shown as red spheres.

UL16 primarily engages MICBpf via a predominantly hydrophobic, glycan-free (see also Ref. [29]) surface comprised of its DEB β-sheet and the adjacent β-strand A, with additional contacts provided by the DE-loop (connecting β-strands D and E) and four amino acids (aa 160–163) at the C-terminus (Figures 3B and 4). This surface interacts with the two long parallel helices at the top of the MICB platform domain and the β5β6-loop connecting β-strands β5 and β6 of MICB (Figures 3A and 4), shielding an area of 2194 Å^2^ from solvent. With the exception of the MICB region that corresponds to the peptide-binding groove in MHC class I proteins, the contact area contains few interfacial solvent-filled cavities. The complex features good surface complementarity (Sc = 0.77) and is highly curved (planarity = 4.0) [38],[39]. Its overall organization resembles a saddle with two stirrups (UL16) that is mounted on horseback (MICBpf) (Figure 4A, see also Figure 1B). The saddle is formed by the DEB β-sheet, whereas the stirrups are contributed by the DE-loop and the C-terminus on either side of the sheet. To facilitate discussion of interactions, we have divided the UL16-MICB interface into three regions (A, B and C, Figure 4). **Contact region A**, which is located at the center of the complex and mostly hydrophobic in nature, contributes 54% of the total contact area. Interactions predominantly involve residues within the DEB β-sheet and β-strand A of UL16. Eight UL16 residues (Trp54, Leu56, Met59, Ile61, Ile63, Tyr125, Leu110 and Leu114) define a compact hydrophobic face that interacts with non-polar regions of MICBpf residues in its central α3-helix. These interactions are augmented with a salt bridge between UL16 Asp112 and MICB Lys152 and a number of mostly water-mediated hydrogen bonds (Figure 4B). **Contact region B**, with 23% of the total contact area, is located at one end of the DEB β-sheet and within the DE-loop of UL16. UL16 residues in this region contact several acidic residues (Glu64, Asp65 and Glu68) in the α1-helix of MICBpf, mostly via polar interactions (Figure 4C). **Contact region C**, which contributes 23% to the total contact area, is located on the other side of the UL16 saddle. Here, the C-terminus of the UL16 ectodomain interacts with the β5β6-loop and the N-terminus of MICBpf via a mixture of hydrophilic and hydrophobic contacts (Figure 4D). The overall architecture of the complex, with its large contact area and substantial number of interactions between contacting residues, indicates tight binding, which is in agreement with our SPR data that place the affinity of UL16 for MICBpf at 66 nM (Figure 2 and Table S1).

### Comparison with the NKG2D-MICA complex

**Figure 5:**
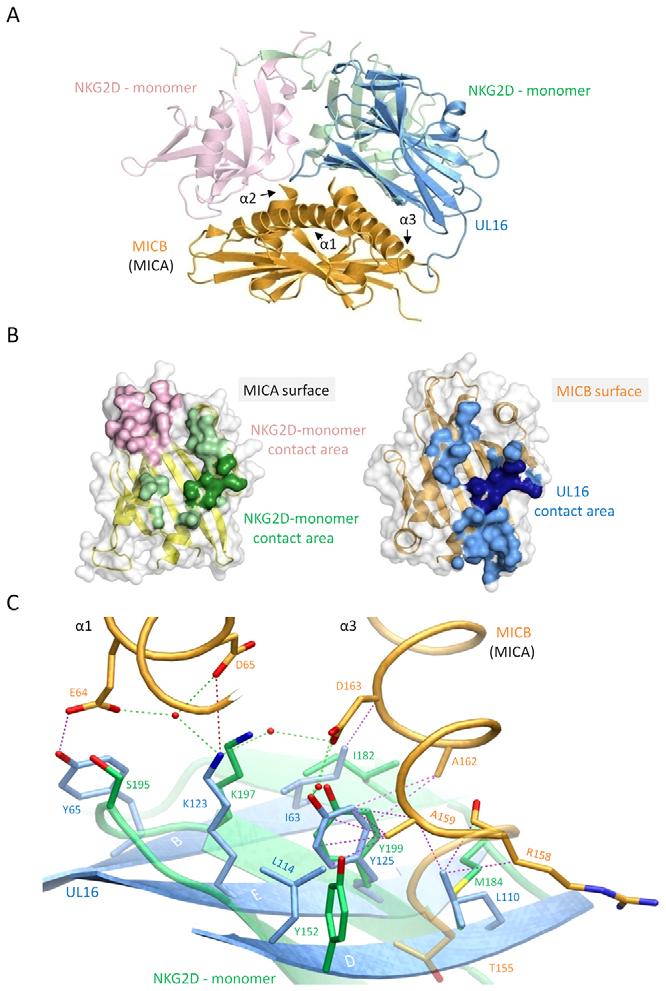
Comparison of the UL16-MICBpf and NKG2D-MICA complex structures. In all panels, the two NKG2D monomers are shown in salmon and green, whereas UL16 and MICBpf are colored blue and orange, respectively. (A), Superposition of the UL16-MICBpf complex onto the MICA-NKG2D complex [26]. MICA, which is very similar to MICB, is not shown for clarity. (B), Ribbon drawings of the α1α2-platform domains of MICA (left side, yellow) and MICB (right side, orange), with their molecular surfaces outlined in grey. Surface-exposed areas of residues that are buried upon complex formation with NKG2D and UL16, respectively, are colored using the color scheme of panel (A). MICB/MICA residues 155, 158, 159, 162 and 163, which contact both UL16 and NKG2D in a similar manner are shown in darker green and blue shading, respectively. (C), Structural mimicry of UL16. Close-up view of the core region of the structures shown in panel (A) with UL16 residues Ile63, Lys123, Tyr125, Leu110, Leu114 that superimpose with chemically equivalent NKG2D residues Ile182, Lys197, Tyr199, Met184 and Tyr152. Side chain atoms, UL16-MICBpf contacts, and water molecules are colored as described in the legend to Figure 4.

A crystal structure of the NKG2D homodimer bound to MICB is unavailable. However, the NKG2D structure in complex with the highly homologous MICA protein [26] shows that both NKG2D monomers make extensive contacts with the long helices at the top of the MICA α1α2-platform domain. The NKG2D-MICA complex buries a surface area of 2170 Å^2^, which is almost exactly the same area buried in the UL16-MICBpf complex. A superimposition of the two complexes demonstrates that contacts formed by UL16 overlap substantially with those made by one NKG2D monomer (Figures 5A, B). One could therefore envision a scenario in which UL16 acts as a direct competitor for NKG2D [18], perhaps even displacing it from its ligands. While the higher affinity of UL16 for MICB and ULBP1 (K_D_ values of 66 and 12 nM, respectively) (Figures 2 and S1 and Table S1) compared with the respective affinities of NKG2D for the same ligands (K_D_ values of 0.8 and 1 µM, respectively)[40] would support this scenario, most reports to date indicate that UL16 acts inside the cell and is therefore unlikely to compete with NKG2D for ligand binding [2],[17],[20],[21].

The detailed comparison of the central contact regions in each case reveals that, despite having entirely different folds, NKG2D and UL16 use an almost identical pattern of amino acid side chains to engage their ligands (Figure 5C, see also Figures 3B and S2). In UL16, this pattern includes the MICB-contacting residues Ile63, Lys123 and Tyr125, while NKG2D uses an identical pattern of residues, Ile182, Lys197, and Tyr199, to form very similar contacts with MICA. Remarkably, although the three side chains are contributed by different structural elements in each case, their position in space overlaps closely (Figure 5C). This is also true for two additional UL16 residues, Leu110 and Leu114, which are hydrophobic in nature and overlap with chemically related NKG2D residues Met184 and Tyr152 (Figure 5C). Together, the five residues constitute a predominantly hydrophobic binding motif that is common to NKG2D and UL16 (Figures 5B, C), and that forms the center of the interaction with the MIC molecules. This central binding motif is augmented by additional contacts, such as those mediated by UL16 residue Tyr65 and NKG2D residue Ser195, that perform similar functions in the UL16-MICBpf and NKG2D-MICA [26] complexes (Figures 3B and 5C). Since all MICA and MICB residues contacted by this central binding motif are identical, and since the structures of MICA and MICB superimpose well in this region, we conclude that UL16 mimics a key structural motif of NKG2D with an entirely different fold in order to engage MICB. Furthermore, we consider it likely that the central binding motif of UL16 also plays an important role in the recognition of other NKG2D ligands, for which structures of complexes with UL16 are not yet available.

## Discussion

Bacterial and viral pathogens often interfere with cellular activities and immunosurveillance processes to enhance their survival and effectiveness [41]. This is typically achieved by virulence factors, which imitate the function of a host protein by mimicking its key structural features. In the majority of such cases, pathogens first hijack and then manipulate host genes to produce structurally homologous versions of host proteins [41]–[45]. Thus, virulence factors and host proteins are derived from the same origin and arise from divergent evolution. However, structural mimics can also be generated through convergent evolution. Although differing in evolutionary origin and three-dimensional structure, the virulence factors have in this case evolved to mimic key structural features of cellular proteins. Examples for the latter strategy, which can only be revealed through structural analyses, are still exceedingly rare and are limited to a small number of virulence factors [41],[46],[47]. The comparison of HCMV UL16 with human NKG2D, reveals a striking example of convergent evolution [41]. A set of five predominantly hydrophobic core residues on the UL16 surface precisely mimics a set of five equivalent residues in the central region of the interface used by the structurally unrelated immunoreceptor NKG2D to interact with its ligands.

As this central binding motif represents only a portion of the total interface between NKG2D and its ligands (Figure 5), one may wonder why UL16 mimics just this particular structural motif of NKG2D. McFarland et al. reported that residues constituting this motif (Tyr152, Met184 and Tyr199) form the basis for the highly degenerate ligand recognition mode of NKG2D [40],[48]. They proposed a “rigid adaptation” mechanism, in which a rigid binding site on NKG2D uses the same set of predominantly hydrophobic core residues to make diverse interactions with a series of chemically and structurally distinct ligand residues. As an example, Tyr199 and Tyr152 of NKG2D can accommodate residues as diverse as Ala, Met or Phe at ligand position 159 [40] (Figures 3A and S2). Mimicry of these core residues likely enables UL16 to employ this binding mechanism of NKG2D to contact a similar set of ligands. The “rigid adaptation” concept is furthermore supported by the finding that UL16 engages its ligands via a rigid β-sheet, which does not allow for much conformational flexibility. The ligand residues contacted by NKG2D and UL16 in MICA and MICB, respectively, are Asp65, Thr155, Ala159, Ala162, Asp163 and the hydrophobic portions of the Arg/His158 side chain (Figures 3A and 5C and S2) [26],[40],[48]. Since NKG2D and UL16 both evolved the same central binding motif in order to contact this specific set of ligand residues, the latter likely represent binding hot spots in MICA and MICB [48]. Furthermore, these residues probably are also of major importance for interactions with ULBP molecules (Figures 3A and S2). We note for instance that (1) based on the “rigid adaptation” concept the amino acid at ligand position 159 can be quite variable in size and chemical nature, (2) Asp163 is conserved in all NKG2D ligands, and (3) alanine and glycine dominate at position 162.

Unlike NKG2D, UL16 engages only MICB, ULBP1, ULBP2 and ULBP6, but not MICA, ULBP3, ULBP4 and ULBP5 [18]–[20], [29]–[31]. Our SPR measurements show that UL16 binds MICB with high affinity, whereas the affinity of UL16 for MICA is negligible (Table S1), in line with earlier studies [2],[21],[29]. Given the high degree of similarity between MICA and MICB at the sequence and structural level, the inability of UL16 to engage MICA is puzzling. In order to better understand the structural parameters that guide UL16 binding to MICB vs. MICA, Spreu et al. [29] assayed binding of soluble UL16-Fc to MICB chimeras in which they had exchanged domains, subdomains and single amino acids of MICB against equivalent regions of MICA. These experiments clearly demonstrated that recognition by UL16 is linked to residues projecting from the helical structures in the MICB α2-domain. However, the molecular mechanism by which these residues confer selectivity remained unclear.

The crystal structure of the UL16-MICB complex now allows us to identify the key determinants of NKG2D ligand binding to UL16. Our structural alignment of MICA and MICB identifies only seven MICB residues that contact UL16 in the complex and that are replaced by other amino acids in MICA (Figure 3A). Residues at positions 64, 71, 75, 102 and 158 can assume alternate conformations that would not interfere with binding, and could in some cases even mediate favorable contacts with UL16. Therefore, their effect on UL16 binding is likely to be negligible (see also Ref. [29]). Replacement of α1-domain Glu68 with glycine (Figure 4C) in MICA would eliminate several hydrophobic contacts and three hydrogen bonds with UL16 residues 117 and 118, and could therefore conceivably have a negative effect on UL16 binding. However, as complete replacement of the α1-domain of MICB by MICA (including residue Glu68) did not significantly affect UL16 binding [29], residue 68 is probably not a key determinant of UL16 binding.

**Figure 6:**
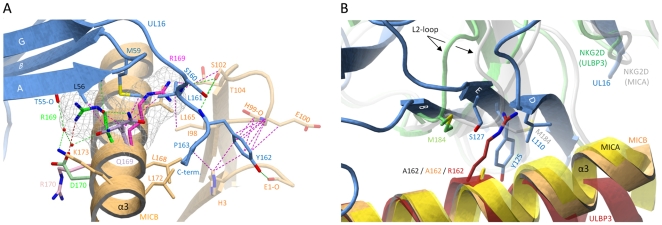
Selectivity of NKG2D ligand binding by UL16. (A), The α1α2-platform domain of NKG2D-bound MICA [26] was superimposed onto MICBpf, but only the MICA side chains Arg170 (pink) and Arg169 (magenta) are shown. The α1α2-domain of NKG2D-bound ULBP3 [28] was also superposed onto MICB, and only the ULBP3 side chains of Arg169 (green) and Asp170 (light green) are shown. Cages surrounding the two arginines of MICA and ULBP3 at position 169 depict the area that these side chains would require in a space-filling model. In both cases, the arginine side chains would clash with UL16 residues. (B), The α1α2-platform domain of NKG2D-bound MICA [26] (yellow) and ULBP3 [28] (red), respectively, was superimposed onto MICBpf (orange). The side chains of alanine (present in MICBpf and MICA) and arginine (present in ULBP3) at position 162 are shown. Also shown are the Met184 side chains of both the MICA-bound (white) and ULBP3-bound (green) NKG2D monomers, both of which correspond to the green NKG2D monomer in Figures 5A and 5B. Conformational changes of the L2-loop of MICA-bound NKG2D displaces Met184 and allows for the accommodation of Arg162 in ULBP3-bound NKG2D. In UL16, the rigid DEB sheet does not allow for a similar conformational adjustment, and ULBP3 residue Arg162 would therefore clash with UL16 residues.

On the other hand, however, Gln169 in the α2-domain of MICB is likely to be critical. Our structure shows that substitution of Gln169 with arginine, which is present at this position in MICA, would lead to steric clashes with UL16 residues Met59 and Leu161 (Figure 6A) that would prevent binding. This is in perfect agreement with previous experiments demonstrating that MICB carrying a Gln169Arg substitution no longer bound UL16 [29]. We consider it in fact likely that the side chain at position 169 is not only the key determinant of selective UL16 binding to the MIC molecules but all NKG2D ligands, which is based on the following reasons. (1) All NKG2D ligands that carry a glutamine or glutamate at position 169, i. e. MICB, ULBP1, ULBP2 and ULBP6, bind UL16, while all ligands that have an arginine at this position, i. e. MICA, ULBP3 and ULBP4, do not bind UL16 (Figure 3A). Although ULBP5 also carries a glutamate at position 169 and should therefore bind UL16, Wittenbrink et al. demonstrated by mutational studies that a substitution in the α2-domain, which is unique among all NKG2D ligands (Figure 3A), prevents binding of ULBP5 to UL16 [31]. (2) Arg169 has a similar conformation, stabilized by contacts with surrounding hydrophobic residues, in the unliganded [27] and liganded [26] MICA structures (Figure 6A). In this orientation, however, the Arg169 side chain would clash with UL16 residues. Modeling suggests that the arginine side chain could adopt only a single rotamer conformation, sandwiched between the hydrophobic side chain regions of Leu172 and Lys173, that would not result in steric clashes with UL16 (Figure 6A). However, such a rotamer is only seen in 2% of all observed arginines [49]. (3) The conformation of Arg169 in the ULBP3 structure [28], which is held in place by a salt bridge to Asp170, would also clash with UL16 (Figure 6A). A similar arrangement of Arg169 can be expected for ULBP4, where Asp170 is replaced with glutamate (Figure 3A). We note that Arg169 is not located near the NKG2D binding site and therefore does not play a role in the interaction of either MICA or ULBP3 with NKG2D.

A second important requirement for binding of NKG2D ligands to UL16 is the presence of a small hydrophobic side chain at position 162. In the UL16-MICBpf complex, Ala162 faces towards Tyr125, a UL16 footprint residue (Figures 5C and 6B). With the exception of ULBP3, which has an arginine at this position, all other NKG2D ligands have either an alanine or a glycine at position 162 (Figures 3A and S2). The long and positively charged Arg side chain of ULBP3 would clash with several UL16 residues (Figure 6B), likely contributing to the failure of UL16 to bind ULBP3 [2],[17],[18],[21] (Table S1). Interestingly, Arg162 would also clash with Met184 of the L2-loop of NKG2D in its MICA-liganded form. To allow for ULBP3 binding, NKG2D undergoes a conformational adjustment in which the L2-loop displaces Met184, resulting in sufficient space for the accommodation of Arg162 (Figure 6B). However, the rigid DEB β-sheet of UL16, which would not allow for such larger conformational adjustments, is unlikely to accommodate Arg162.

Taken together, these analyses suggest that some NKG2D ligands apparently bypass intracellular retention by UL16 through alteration of a small number of key residues at strategic locations of their potential UL16 binding interface. We therefore consider it likely that the selective pressure exerted by UL16 contributed to drive the diversification of NKG2D ligands, which eventually may have led to the emergence of non-UL16 binding ligands such as MICA and ULBP3 [2],[5],[17],[18],[23]. Further support for an HCMV-driven diversification of NKG2D ligands comes from studies by Cosman and colleagues showing that the HCMV immunoevasin UL142 targets most MICA allelic variants except MICA*08 [2],[5],[22]. Intriguingly, MICA*08 contains a truncated cytoplasmic domain and is by far the most frequent MICA variant in many populations [22]. As yet, no direct interaction of UL142 and MICA has been shown and the molecular mechanisms of MICA sequestration by UL142 are unknown. In contrast to UL16, UL142 and MCMV-encoded immunoevasins m145, m152, and m155 that suppress surface expression of mouse NKG2D ligands MULT-1, RAE-1, and H60, respectively, are predicted to have an MHC class I-like fold [2], [22], [23], [50]–[54]. It will be of great interest to determine the structural basis of NKG2D ligand engagement by MHC class I-like HCMV immunoevasins and to compare these interactions of two MHC class I-like molecules to those of the NKG2D-like ligand binding mode of UL16.

NK receptors binding to MHC class I or class I-like molecules belong to two structurally distinct families, the Ig superfamily and the C-type lectin superfamily [10]. While NKG2D belongs to the latter group, our structural analysis shows that UL16 assumes an Ig-like fold. Therefore, one may ask whether UL16 is related to the Ig-like NK receptors that bind MHC class I molecules, such as the leukocyte Ig-like receptors (LIRs) or the killer immunoglobulin-like receptors (KIRs). Structures of LIR-1 in complex with HLA-A2 [10] and with the HCMV MHC class I decoy UL18 [45] show that, in both cases, LIR-1 contacts β2-microglobulin and the α3-domain of the HLA-A2 and UL18 ligands via loops located at the interdomain hinge region of its two tandem Ig domains. In contrast KIRs, like UL16, engage the α-helical parts of the platform domain of MHC class I molecules, but, similar to LIRs, employ loops at the interdomain hinge region of their Ig domains for this interaction [10]. Therefore, LIRs and KIRs exhibit an MHC class I-binding mode that is distinct from that used by UL16. Since there is also no obvious sequence homology between these Ig-like NK receptors and UL16, we favor the view that UL16 evolved independently, mimicking a central binding motif of the structurally unrelated NKG2D immunoreceptor.

To the best of our knowledge, the structure presented here is the first structure of a viral immunoevasin in complex with a stimulatory NK receptor ligand as well as the first reported case of structural mimicry through convergent evolution of a human immunoreceptor by a viral immunoevasin. The results of our structural analyses revealed that HCMV and humans independently evolved two structurally distinct receptors, NKG2D and UL16, that share the same central ligand binding motif in order to achieve promiscuous binding to MIC and ULBP molecules. Our findings provide new insights into the structural basis of the evolutionary struggle between persistent viruses and cellular immune surveillance, exemplified by the promiscuous binding mode of the HCMV immunoevasin UL16 and the diversification of NKG2D ligands.

## Materials and Methods

### Expression and purification

#### Expression and purification of UL16

A recombinant cDNA fragment including the N-terminal signal peptide (residues 1–26) and the ectodomain (residues 27–184) of UL16 (GI: 9625700; UniProt P16757) was fused to a thrombin cleavage site followed by the human IgG1-Fc sequence and cloned into a pcDNA3.1^©^(−) vector (Invitrogen). CHO Lec 3.2.8.1 cells [34] were stably transfected with this construct using Lipofectamine 2000 (Invitrogen), and selected with 1.5 mg/ml G418 (Invitrogen) in α-MEM, supplemented with 10% Ultra-Low IgG FCS (Invitrogen), 100 U/ml penicillin (PAA), 100 µg/ml streptomycin (PAA), 2 mM L-glutamine (Invitrogen), and 1 mM pyruvate (PAA). A single cell clone was selected and grown in roller bottles at 37°C and 5% CO_2_. For each purification, 10 liters of cell culture supernatant were filtered through a 0.2 µm filter, adjusted to pH 9 with 5 M NaOH, and loaded overnight onto two serially connected Protein A HP columns (5 ml column volume each; GE Healthcare) using an Äkta FPLC system (GE Healthcare). The columns were extensively washed with Protein A binding-buffer (500 mM NaCl, 170 mM glycine pH 9 at 4°C) and the protein eluted with arginine-buffer (10 mM NaCl, 500 mM arginine pH 4.1 at 4°C) directly into reservoir-buffer (500 mM HEPES pH 9 at 4°C). Fractions containing UL16 were pooled, dialysed against TBS (pH 8 at 22°C), and concentrated. Thrombin (Sigma) cleavage was performed with 1 U/mg recombinant protein at 22°C for 18 hours. The cleaved samples were diluted five-fold in Protein A binding-buffer and run over two consecutive Protein A HP columns (1 ml column volume each; GE Healthcare) followed by a benzamidine column (1 ml column volume; GE Healthcare) to remove cleaved Fc tag and thrombin, respectively. Flow-through containing UL16 was concentrated and dialyzed against TBS (pH 7.4 at 4°C) for storage. UL16 migrated as a single band on reducing SDS gels, but appeared as two bands, corresponding to monomers and dimers, on non-reducing gels. Dimeric UL16 did not bind MICB and could not be converted to monomer by incubation with reducing agents. The dimer was separated from the monomer with hydrophobic interaction chromatography (HIC). Briefly, the UL16 monomer/dimer mixture was diluted in HIC-binding buffer (1 M NaCl, 0.05 M Na_2_HPO_4_ pH 7.4 at 4°C), loaded onto 3 serially connected phenyl sepharose columns (5 ml column volume each; GE Healthcare), and washed extensively with HIC-binding buffer. Phenyl-sepharose bound monomer was eluted with HIC-elution buffer (0.05 M Na_2_HPO_4_ pH 7.4 at 4°C) and dialysed against TBS pH 7.4 at 4°C. The yield of monomeric UL16 was 0.2 mg per 1 liter of cell culture supernatant.

#### Expression and purification of MICB

Gene sequences (GI:2454261; UniProt Q29980) encoding ectodomains α1 and α2 of MICB*02 (MICBpf, residues 1–181) and ectodomains α1, α2 and α3 of MICB*02 (residues 1–276) were fused to a thrombin cleavage site followed by (His)_8_- and (His)_6-_tags, respectively, and cloned into the pET-21a(+) vector (Novagen). Both proteins were purified with the same strategy. *E.coli* Rosetta2 cells transformed with the appropriate expression construct were grown in LB medium, supplemented with 50 µg/ml ampicillin and 34 µg/ml chloramphenicol, to an optical density (OD)_600_ of 0.6 before induction with 1 mM IPTG at 37°C for 12 hours. Inclusion bodies containing MICB were refolded by stepwise arginine/urea dialysis. Soluble MICB in TBS (pH 7.4 at 4°C) was further purified via Ni-NTA affinity chromatography (1 ml column volume; GE Healthcare) followed by Superdex 75 size-exclusion chromatography (GE Healthcare).

#### Complex formation

UL16 and an excess amount of MICBpf were incubated for 16 hrs in TBS pH 7.4 at 4°C. The complex was separated from excess MICB through gel filtration (Superdex 75). In order to obtain diffracting crystals, UL16 was deglycosylated by incubation with EndoH after complex formation. Briefly, complex was diluted in EndoH-buffer (0.1 M NaAc pH 5.2 at 25°C), containing 0.5 U/µl EndoH (NEB) and incubated for 1 h at 37°C. Removal of cleaved glycans and EndoH was performed by size exclusion chromatography (Superdex 75). The complex was then concentrated to 15 mg/ml and used for crystallization.

### Surface plasmon resonance

All SPR experiments were performed and evaluated as described previously [55]. Using two consecutive flow cells on a CM5 biosensor chip, MICBα1α2 (MICBpf) and MICBα1α3 ligands, respectively, were each covalently immobilized on the surface of the downstream (experimental) flow cell via amine-coupling chemistry (GE Healthcare) following manufacturer's instructions, while the surface of the upstream (reference) flow cell was subjected to the same coupling reaction in the absence of protein. For the Protein A-G chip preparation, an amount of 3500 RU (resonance units) of recombinant Protein A-G (BioVision) was covalently immobilized to the upstream (reference) and downstream (experimental) flow cells of a CM5 biosensor chip (GE Healthcare) by amine-coupling chemistry (GE Healthcare). Fc-tagged ULBP1, ULBP2, ULBP3 (all R&D Systems), ULBP4 and ULBP5 ligands [31] were diluted in HBS-EP (10 mM HEPES, 150 mM NaCl, 3 mM EDTA, 0.005% (v/v) Surfactant P20, pH 7.4 at 25°C) and noncovalenty bound to the experimental flow cell surface. In all experiments, untagged, monomeric UL16 analyte was serially diluted in running buffer and injected in series over the reference- and experimental biosensor surface at 50 µl/min. After each cycle using a Protein A-G chip, the biosensor surface was regenerated (stripped of any remaining analyte and ligand) with two 1 min injections of 10 mM glycine pH 1.7. CM5 chips were not regenerated.

### Crystallization

For crystallization, complex at 15 mg/ml was mixed in a 1∶1 ratio with a reservoir solution containing 0.2 M ammonium sulfate, 0.1 M sodium cacodylate pH 6.5, and 25% PEG 8000. Crystals grew at 4°C over a time period of 4 months using the hanging drop vapor diffusion method. They were soaked in reservoir solution enriched with 15% ethylene glycol, and then flash frozen in liquid nitrogen prior to data collection.

### Structure determination

The crystals belong to space group P2_1_2_1_2_1_ and contain two complexes in the asymmetric unit. All diffraction data were collected at 100 K and a wavelength of 1.0013 Å at the Swiss Light Source (SLS, Villigen, Switzerland) beamline X06SA using the PILATUS 6M detector. Data were indexed, integrated and scaled with XDS [56], and the structure was solved by molecular replacement as implemented in PHASER [57] using the unliganded MICB structure [25] (PDB code 1JE6) as search model. The initial density map already clearly showed the approximate location of the UL16 molecules. Phases were then improved through non-crystallographic symmetry averaging using RESOLVE [58]. Structural refinement was performed with PHENIX [59] and model building was done with Coot [49]. Refinement included TLS-refinement of 26 TLS groups assigned by the TLSMD Server [60]. A data set for R_free_ calculation was generated with 5% randomly selected reflections, and refinement progress was monitored by the decrease of R and R_free_ throughout. The final model has R and R_free_ values of 17.7% and 21.5%, respectively, and was validated using PROCHECK [57] and WHAT_IF [61]. Secondary structure elements were assigned with DSSP [62]. Structural figures were created with PyMOL [63].

### Glycan modeling

Eight potential N-linked glycosylation sites were identified in the UL16 ectodomain. Six of the possible eight asparagine residues (Asn41, 68, 84, 95, 101, 132) carry NAG residues that are clearly defined by electron density (Figure 1A). While extra density is present at the seventh residue, Asn35, this density is not well defined, and no NAG residue was built at this location. No extra electron density is observed at the final asparagine, Asn145, and thus this residue is either not glycosylated or carries an especially flexible glycan moiety. We note that Asn145 is close in space to Asn35, which is glycosylated. In order to produce a realistic estimate of size and distribution of the glycan structure of native UL16 (Figure 1B) we used the GlyProt [64] online server and modeled hybrid and complex glycans linked to the seven Asn residues with NAG electron density.

### Accession numbers

Atomic coordinates and structure factors have been deposited with the Protein Data Bank under accession code 2wy3.

## Supporting Information

Table S1 Kinetic and affinity data determined by SPR
(0.04 MB DOC)

Figure S1 Schematic view of the structural mimicry of UL16. The blue regions highlight the five UL16 and NKG2D footprint residues participating in structural mimicry. UL16 residues are shown in white, the corresponding NKG2D residues are shown in black. MICA and MICB residues that are contacted by the footprint are placed in yellow circles, at the approximate position of interaction. Also shown are the amino acids at corresponding positions in ULBP1, ULBP5/6, ULBP2, ULBP3 and ULBP4. In ULBP3 [28], a kink in the α3-helix starting at position 162 (Figure 6B) causes a one-residue shift towards the N-terminus. In these cases, the shifted ULBP3 residue that corresponds to the MICBpf residue is given by a superscript number following the ULBP3 one letter code. As an example, ULBP3 position Met168 and not Val169 corresponds to MICBpf position Ala159. Also as a result of the helix kink, no ULPB3 residue corresponds in space to the MICBpf residue in position 155, indicated by (#). Interactions between residues are represented with arrows, accompanied by green text for hydrogen bonds, red text for salt bridges, and magenta text for hydrophobic contacts; the blue text indicates the clash of ULBP3 Arg162 (Figure 3A) with Leu100 of UL16 or Met184 of NKG2D as observed in the MICA/NKG2D complex structure [26] (Figure 6B).
(0.96 MB TIF)

Figure S2 Schematic view of the structural mimicry of UL16. The blue regions highlight the five UL16 and NKG2D footprint residues participating in structural mimicry. UL16 residues are shown in white, the corresponding NKG2D residues are shown in black. MICA and MICB residues that are contacted by the footprint are placed in yellow circles, at the approximate position of interaction. Also shown are the amino acids at corresponding positions in ULBP1, ULBP5/6, ULBP2, ULBP3 and ULBP4. In ULBP3 [28], a kink in the α3-helix starting at position 162 (Figure 6B) causes a one-residue shift towards the N-terminus. In these cases, the shifted ULBP3 residue that corresponds to the MICBpf residue is given by a superscript number following the ULBP3 one letter code. As an example, ULBP3 position Met168 and not Val169 corresponds to MICBpf position Ala159. Also as a result of the helix kink, no ULPB3 residue corresponds in space to the MICBpf residue in position 155, indicated by (#). Interactions between residues are represented with arrows, accompanied by green text for hydrogen bonds, red text for salt bridges, and magenta text for hydrophobic contacts; the blue text indicates the clash of ULBP3 Arg162 (Figure 3A) with Leu100 of UL16 or Met184 of NKG2D as observed in the MICA/NKG2D complex structure [26] (Figure 6B).
(0.45 MB TIF)
