# Supplementary material for: Structure of the HCMV UL16-MICB Complex Elucidates Select Binding of a Viral Immunoevasin to Diverse NKG2D Ligands
Source: PLoS Pathog. 2010 Jan 15;6(1):e1000723. doi: 10.1371/journal.ppat.1000723 (PMC2797645; doi:10.1371/journal.ppat.1000723)
Supplement: Table S1 — Kinetic and affinity data determined by SPR (0.04 MB DOC) [file ppat.1000723.s001.doc]

**Supplemental Table S1**

**Table S1: Kinetic and affinity data determined by SPR**

| Experimental setup | | Steady-State-Analysis | Kinetic Analysis | | | Average Affinity |
| --- | --- | --- | --- | --- | --- | --- |
| Immobilized  (ligand) | in solution  (analyte) | K_D_ [M] | K_D_ [M] | k_on_ [M^-1^s^-1^] | k_off_ [s^-1^] | K_D_ [M] |
| MICBpf (α1-2) | UL16 | 66∙10^-9^ | 66∙10^-9^ | 0.82∙10^6^ | 0.054 | 66∙10^-9^ |
| MICB (α1-3) |  | 68∙10^-9^ | 66∙10^-9^ | 0.68∙10^6^ | 0.045 | 67∙10^-9^ |
| ULBP1-Fc |  | n.d.* | 12∙10^-9^ | 1.31∙10^6^ | 0.016 | 12∙10^-9^ |
| ULBP2-Fc |  | 43∙10^-9^ | 42∙10^-9^ | 1.71∙10^6^ | 0.072 | 43∙10^-9^ |
| ULBP3-Fc |  | No SPR response was observed upon injection of 100 µM UL16^a)^ | | | | |
| ULBP4-Fc |  |  |  |  |  |  |
| ULBP5-Fc |  |  |  |  |  |  |

^a)^ Samples with low analyte concentrations did not reach chemical equilibrium (plateau phase) during injection, which is required to perform a reliable steady-state analysis.
