# Supplementary figures and images for: Structure of the HCMV UL16-MICB Complex Elucidates Select Binding of a Viral Immunoevasin to Diverse NKG2D Ligands
Source: PLoS Pathog. 2010 Jan 15;6(1):e1000723. doi: 10.1371/journal.ppat.1000723 (PMC2797645; doi:10.1371/journal.ppat.1000723)

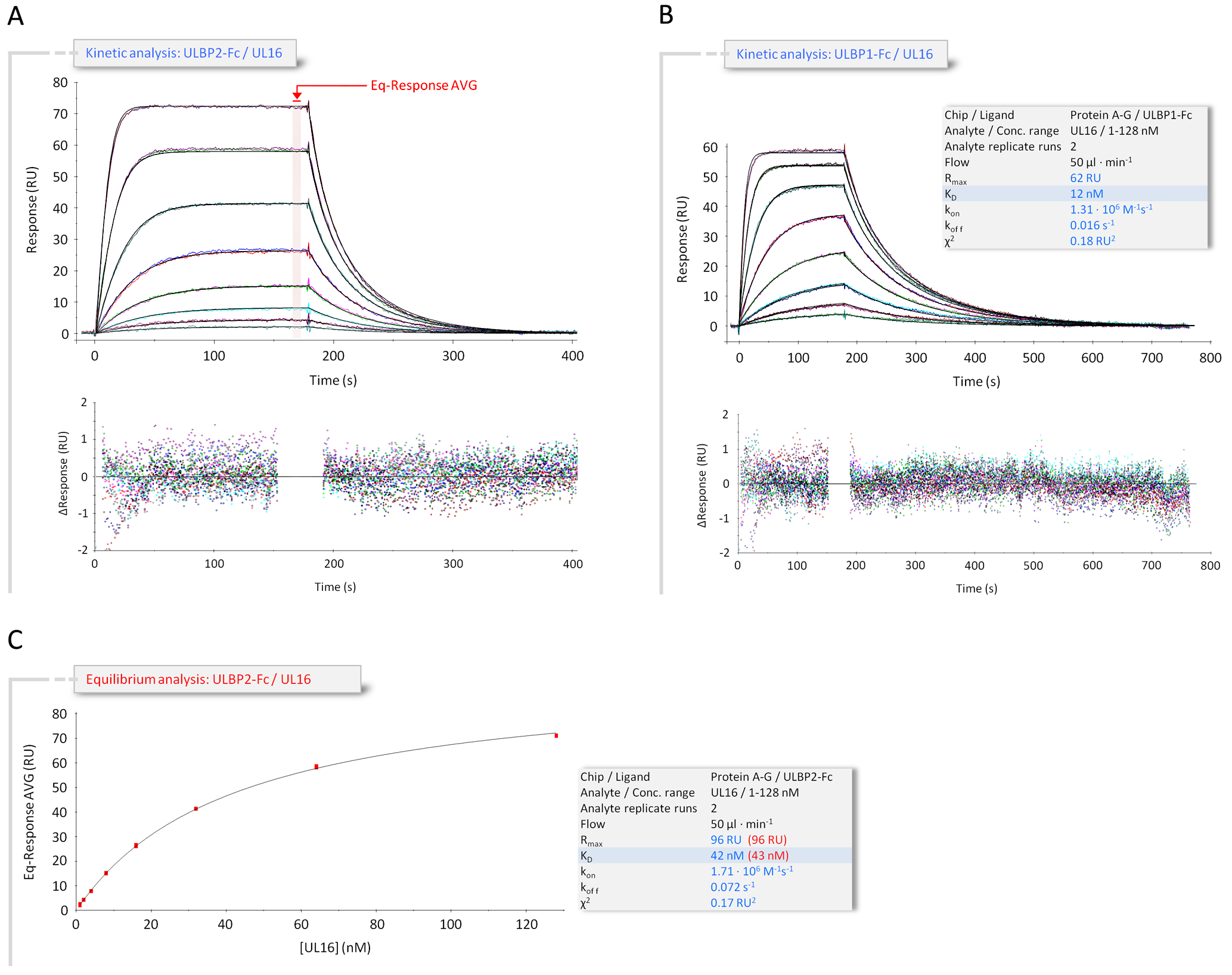

Supplement: Figure S1 — Schematic view of the structural mimicry of UL16. The blue regions highlight the five UL16 and NKG2D footprint residues participating in structural mimicry. UL16 residues are shown in white, the corresponding NKG2D residues are shown in black. MICA and MICB residues that are contacted by the footprint are placed in yellow circles, at the approximate position of interaction. Also shown are the amino acids at corresponding positions in ULBP1, ULBP5/6, ULBP2, ULBP3 and ULBP4. In ULBP3 [28], a kink in the α3-helix starting at position 162 (Figure 6B) causes a one-residue shift towards the N-terminus. In these cases, the shifted ULBP3 residue that corresponds to the MICBpf residue is given by a superscript number following the ULBP3 one letter code. As an example, ULBP3 position Met168 and not Val169 corresponds to MICBpf position Ala159. Also as a result of the helix kink, no ULPB3 residue corresponds in space to the MICBpf residue in position 155, indicated by (#). Interactions between residues are represented with arrows, accompanied by green text for hydrogen bonds, red text for salt bridges, and magenta text for hydrophobic contacts; the blue text indicates the clash of ULBP3 Arg162 (Figure 3A) with Leu100 of UL16 or Met184 of NKG2D as observed in the MICA/NKG2D complex structure [26] (Figure 6B). (0.96 MB TIF) [file ppat.1000723.s002.tif]

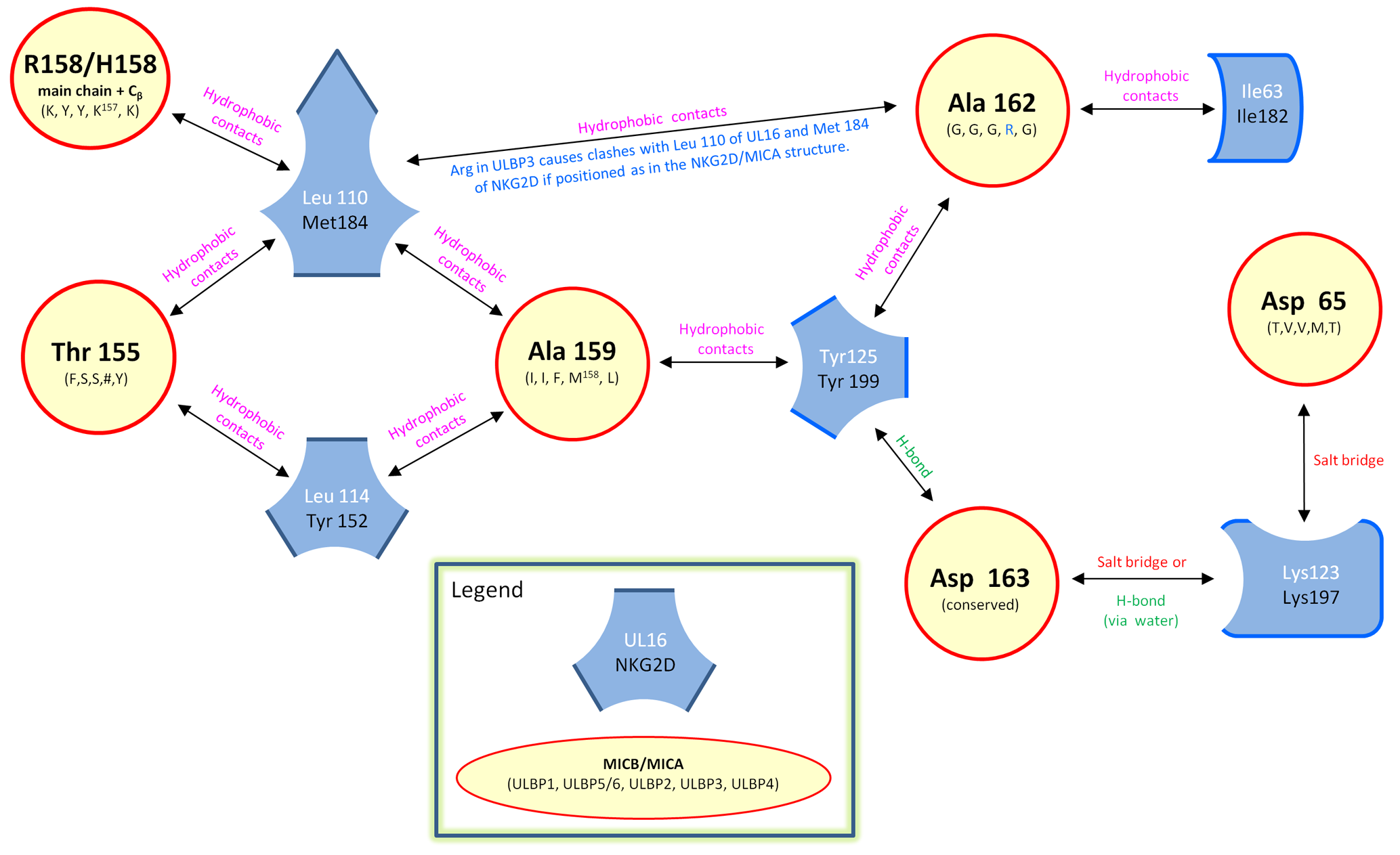

Supplement: Figure S2 — Schematic view of the structural mimicry of UL16. The blue regions highlight the five UL16 and NKG2D footprint residues participating in structural mimicry. UL16 residues are shown in white, the corresponding NKG2D residues are shown in black. MICA and MICB residues that are contacted by the footprint are placed in yellow circles, at the approximate position of interaction. Also shown are the amino acids at corresponding positions in ULBP1, ULBP5/6, ULBP2, ULBP3 and ULBP4. In ULBP3 [28], a kink in the α3-helix starting at position 162 (Figure 6B) causes a one-residue shift towards the N-terminus. In these cases, the shifted ULBP3 residue that corresponds to the MICBpf residue is given by a superscript number following the ULBP3 one letter code. As an example, ULBP3 position Met168 and not Val169 corresponds to MICBpf position Ala159. Also as a result of the helix kink, no ULPB3 residue corresponds in space to the MICBpf residue in position 155, indicated by (#). Interactions between residues are represented with arrows, accompanied by green text for hydrogen bonds, red text for salt bridges, and magenta text for hydrophobic contacts; the blue text indicates the clash of ULBP3 Arg162 (Figure 3A) with Leu100 of UL16 or Met184 of NKG2D as observed in the MICA/NKG2D complex structure [26] (Figure 6B). (0.45 MB TIF) [file ppat.1000723.s003.tif]
